# Quantitative Analysis of Polyphenols and In Vitro Antioxidant, Antimicrobial and Toxicity Assessments in Needles of Five *Pinus* Species from Montenegro

**DOI:** 10.3390/microorganisms14010170

**Published:** 2026-01-13

**Authors:** Alma Kurtiš, Jelena Antić-Stanković, Biljana Bufan, Dragana D. Božić, Slađana Krivokapić, Biljana Damjanović-Vratnica, Svetlana Perović

**Affiliations:** 1 Biology Department, Faculty of Science and Mathematics, University of Montenegro, 81000 Podgorica, Montenegro; kurtisalma@gmail.com (A.K.); sladjana.krivokapic@gmail.com (S.K.); 2 Department of Microbiology and Immunology, Faculty of Pharmacy, University of Belgrade, 11221 Belgrade, Serbia; jelena.stankovic@pharmacy.bg.ac.rs (J.A.-S.); biljana.bufan@pharmacy.bg.ac.rs (B.B.); dragana.bozic@pharmacy.bg.ac.rs (D.D.B.); 3 Faculty of Metallurgy and Technology, University of Montenegro, 81000 Podgorica, Montenegro; biljanad@ucg.ac.me

**Keywords:** conifers, pine needles, extraction methods, biological potential, secondary metabolites

## Abstract

This study aimed to investigate the chemical composition and biological potential of needle extracts from five pine species, including antimicrobial, antioxidant, and cytotoxic activity, as well as their influence on cell cycle progression. Needle extracts were prepared using three extraction methods: conventional maceration (CM), ultrasound-assisted extraction (UAE), and digestion (D). The chemical profile was determined with an emphasis on phenolic acids, flavonoids, and related phenolic compounds. The highest total phenolic content was observed in *Pinus sylvestris* (3.438 mg/g GAE), followed by *Pinus heldreichii* (2.732 mg/g GAE). Rutin, ferulic acid, and quercitrin were identified as the predominant phenolic compounds. The highest total flavonoid content was found in *Pinus pinea* extracts obtained by digestion (1.213 mg/g QE), followed by *P. heldreichii* (1.074 mg/g QE) and *Pinus halepensis* (1.074 mg/g QE), both obtained by UAE. Among all examined species, *Pinus pinea* exhibited the highest TTC values, regardless of the extraction method (7.31–8.21 mg/g GAE). Antibacterial testing showed that *P. pinea* had an MIC of 19 mg/mL against *Enterococcus faecium*, while *P. sylvestris* had the same MIC against *Bacillus spizizenii*. All extracts exhibited cytotoxic effects using MTT assay against HeLa cells at concentrations of 8%, 16%, and 32%, while LS 174T cells were the least sensitive. Pine needle extracts from Montenegro are a valuable source of phenolic and flavonoid compounds, and they demonstrate antimicrobial and cytotoxic activities. The results support the need for further in vivo studies and elucidation of mechanisms of action in order to assess their potential application as novel bioactive agents.

## 1. Introduction

The use of plants in the prevention and treatment of numerous human diseases dates back to ancient times. Archeological findings suggest that plant-derived substances with medicinal properties were known to the Babylonians as early as 6000 BC [1]. Given that plants are capable of producing hundreds or even thousands of metabolites—many of which serve as sources of biologically active compounds and pharmaceutical agents—there is significant scientific interest in their phytochemical profiling and in evaluating their biological potential, including antioxidant, antimicrobial and anti-inflammatory activities.

Polyphenolic compounds—including phenolic acids, flavonoids, anthocyanins, lignans, and stilbenes—along with carotenoids, are key contributors to the bioactivity of plants. These compounds exert protective effects through their antioxidant activity, stimulation of the immune system, regulation of gene expression involved in cell proliferation and apoptosis, and modulation of hormone metabolism. They also exhibit antiviral and antibacterial properties [2,3,4,5,6,7,8,9,10,11,12,13,14,15,16]. Plant-derived polyphenolic compounds (PCs), often referred to as bioflavonoids, are increasingly being used as therapeutic agents in the treatment of diseases with various etiologies [6,17,18].

The increasing interest in natural antioxidants is driven by concerns regarding the safety of synthetic compounds, such as butylated hydroxyanisole (BHA) and tertiary butylhydroquinone (TBHQ), which have been linked to adverse effects, including carcinogenicity [19,20]. Consequently, natural biological resources are being actively investigated for their potential to promote human health and improve quality of life in a sustainable way.

Montenegro is recognized for its rich biodiversity, with approximately 1.2% of the world’s flora and ranking among the leading European regions in terms of plant species density [21]. This rich natural heritage presents significant potential for scientific exploration, particularly in evaluating the chemical composition and biological activity of native plant species for possible applications in pharmaceutical, agricultural, and biotechnological industries.

Recent studies [22,23] have shown that the genus *Pinus* (family *Pinaceae*), comprising over 100 recognized species, is the largest extant genus of conifers.

Due to the scarcity of studies on phenolic-rich extracts of Balkan *Pinus* species, our comparative interpretation necessarily draws on the more abundant essential-oil literature, which remains the most comprehensive source of chemical data for these taxa.

Recent studies from the Balkan region reveal considerable chemical diversity within *Pinus* species, particularly in their volatile and non-volatile metabolites. Essential-oil analyses of *Pinus peuce* from Serbia and Montenegro [24] identified a rich terpenoid profile, while natural populations of *P. heldreichii* in the Scardo–Pindic Mountains Serbia, North Macedonia, Albania) exhibit notable needle morpho-anatomical variation [25]. Bulgarian studies further demonstrate pronounced chemical differentiation among Balkan pines. Terpene-based analyses clearly separate *P. peuce* from *Pinus nigra* and *P. heldreichii* [26], while regional assessments of *P. heldreichii* reveal distinct mountain-specific profiles dominated by limonene and α-pinene [27]. Earlier population studies in Bulgaria also reported distinct terpene profiles among *Pinus heldreichii* stands [28], highlighting pronounced variation in chemical composition across populations.

In contrast, phenolic composition has been less frequently addressed. A study from North Macedonia [29], identified 17 phenolic compounds in *P. peuce* needles, underscoring the relevance of polar metabolites in these taxa. Ecological research complements these findings: palynological evidence shows long-term persistence of *P. peuce* in the southwestern Balkans (Bulgaria, Albania, North Macedonia) [30], while Montenegro hosts a diverse fungal phyllosphere associated with *P. heldreichii* [31].

A recent regional study also highlighted species and population specific variation in needle terpene composition across Balkan *Pinus* taxa, supporting their use in chemotaxonomic differentiation [32].

This study aimed to assess the impact of different extraction methods on polyphenol content and to evaluate the in vitro biological activities of needle extracts from five Montenegrin *Pinus* species.

## 2. Materials and Methods

### 2.1. Plant Material

Fresh needle material was collected from mature trees of five *Pinus* species—*Pinus sylvestris*, *Pinus nigra*, *Pinus heldreichii*, *Pinus halepensis*, and *Pinus pinea*—between January and March 2021. Sampling was conducted at the following locations in Montenegro: Kosanica—Đurđevića Tara (Pljevlja), Virpazar (Bar), the Saddle of Orjen (Herceg Novi), and Mirišta, Luštica Peninsula (Herceg Novi). The geographical positions of the populations were recorded using the GPS Altitude application, version 5.11 (278) (Table 1). A total of 15 samples were analyzed in this study, comprising five *Pinus* species, with needle extracts prepared using three different extraction methods: conventional maceration (CM), ultrasound-assisted extraction (UAE), and digestion (D).

### 2.2. Extraction Procedure

Extraction is the first step in separating the desired natural compounds from the raw materials. To analyze flavonoids, phenolic compounds and other groups of secondary metabolites, samples containing 1.0 g of plant material were processed. Three extraction methods were used: Conventional maceration (CM), ultrasound-assisted extraction (UAE) and digestion (D). The extraction procedures for sample preparation were carried out with 10 mL of 80% methanol (Sigma-Aldrich, St. Louis, MO, USA).

The digestion procedure referred to the next steps: the drug—crushed pine needles (1 g) was poured with 10 mL of solvent (80% methanol) and then extracted in an ultrasonic bath (SONIC Niš, Niš, Serbia) without the presence of ultrasound for 2 h, with occasional mechanical assistance by a glass rod and a constant temperature of 50 °C. The extracts obtained were then centrifuged at 4000 rpm for 20 min.

In the CM procedure, 1 g of crushed pine needles was added to 10 mL of 80% methanol solvent in an oven and crushed with a pestle occasionally for 2 h at room temperature.

During the UAE procedure, 1 g of crushed pine needles was combined with 10 mL of 80% methanol solvent and subjected to ultrasound-assisted extraction in an ultrasonic bath for 30 or 45 min, depending on the requirements of subsequent chemical analyses, which include the determination of total phenols, tannins, antioxidant activity of the extracts, and other related parameters. The extraction was performed at a constant temperature of 50 °C, with continuous ultrasound application. The ultrasonic bath was set to the desired temperature, which was maintained by an integrated thermometer. To ensure accuracy, the temperature was independently verified using an additional thermometer submerged in the water bath.

The extracts were then filtered through filter paper Whatman (Danlab, Podgorica, Montenegro) and stored at a temperature of +4 °C until use.

The obtained methanol extracts were used for the determination of total phenolic, total flavonoid and total tannin content and for the determination of antimicrobial and antioxidant activity as well as for the evaluation of the cytotoxic effect of pine needle extracts on cancer cell lines and the effect on the cell cycle of HeLa cells.

### 2.3. High-Performance Liquid Chromatography (HPLC)

#### HPLC Analysis of Flavonoids, Tannins and Other Phenolic Compounds

The chemical characterization of the examined extract and the quantification of selected compounds were performed before and after hydrolysis using an Agilent Technologies 1100 liquid chromatograph equipped with a diode array detector (Santa Clara, CA, USA). Gallic, p-coumaric, caffeic, quercitrin, and chlorogenic acids were analyzed following a validated HPLC method described by Salaj et al. (2020) [33]. A 10 μL aliquot of pomace extract was injected, and separation was achieved on a reversed-phase Nucleosil C18 column (250 mm × 4.6 mm, 5 μm particle size; Agilent Technologies) maintained at 30 °C. The mobile phase consisted of solvent A (0.1% (*v*/*v*) aqueous HCOOH with 10 mmol CH_3_COONH_4_) and solvent B (pure methanol), delivered in gradient mode (0 min 10% B; 10 min 25% B; 20 min 45% B; 35 min 70% B; 40 min 100% B; 46 min 10% B) using a variable flow rate (0–10 min, 1.0 mL·min^−1^; 10–20 min, 0.8 mL·min^−1^; 20–30 min, 0.7 mL·min^−1^; 30–46 min, 1.0 mL·min^−1^). The total run time was 48 min.

Ellagic acid was quantified using a Zorbax SB-C18 column (250 × 4.6 mm, 5 μm), following an HPLC protocol adapted from Zhou et al. (2008) [34]. A 10 μL aliquot of pomace extract was injected, and the mobile phase consisted of methanol, ethyl acetate, and potassium dihydrogen phosphate: phosphoric acid (both 0.05 M) in the ratio 34:2:64 (*v*/*v*). Ellagic acid was detected at 254 nm using a constant flow rate of 1.0 mL/min at 30 °C.

Regarding *m*/*z* values, these can be obtained using mass spectrometry (MS); however, MS was not applied in this study. Retention times (tR) for all analyzed compounds are provided, with further analytical details available in Salaj et al. (2020) [33] and Zhou et al. (2008) [34].

### 2.4. Determination of Total Phenolic Content (TPC), Total Flavonoid Content (TFC) and Total Tannin Content (TTC)

The total phenolic content (TPC) of the pine needle extracts was determined using the Folin–Ciocalteu reagent (Sigma-Aldrich, St. Louis, MO, USA) according to a method described by Singleton et al. (1999) [35] with slight modifications. The total phenolic content (TPC) of the pine needle extracts is measured using the Folin–Ciocalteu spectrophotometric method. The results of the total phenolic content (TPC) are expressed in milligrams of gallic acid equivalents (GAE) per liter of extract (mg GAE/L), based on the gallic acid standard curve. (y = 0.0032x + 0.0798, R^2^ = 0.9359). Total flavonoid content (TFC) was determined using the aluminum chloride colorimetric method as described by Pękal and Pyrzynska [36], with slight modifications. Quercetin (Sigma-Aldrich, St. Louis, MO, USA) was used to construct the calibration curve (5–120 mg/L). Briefly, 0.4 mL of sample extract was mixed with 2.0 mL of distilled water, 0.3 mL of 5% NaNO_2_, and 0.5 mL of 10% AlCl_3_. After 5 min, 2.0 mL of 1 M NaOH was added, and the mixture was incubated for an additional 6 min. Absorbance was measured at 510 nm. TFC was expressed as mg quercetin equivalents (QE)/mL of extract. The methanol extracts obtained were used for the determination of total phenolic and total flavonoid content and for the analysis and evaluation of antioxidant activity. Total tannin content (TTC) was determined following Würger, McGaw, and Eloff (2014) [37]. Dried, powdered leaves were extracted with acetone, and tannins were quantified using a radial protein-precipitation assay with bovine serum albumin in agar. Results were expressed as gallic acid equivalents (GAE).

### 2.5. Determination of the Antimicrobial Potential of Pine Extracts

The standard microdilution method was used to evaluate the antimicrobial potential of pine needle extracts. The determination of minimum inhibitory concentrations (MICs) was performed using the microdilution method [38]. Gram-positive bacterial strains: *E. faecalis* (29212), *E. faecium* (6057), *B. spizizenii* (6633), *S. aureus* (6538) *and Gram-negative bacterial strains: P. mirabilis* (25933), *E. coli* (25922), *K. aerogenes* (13048), *K. pneumonia* (13883), *P. aeruginosa* (9027), *S. enteritidis* (13076) were used to evaluate the antimicrobial potential of the pine species extracts. All strains used in this study originate from the ATCC collection. All bacterial cultures were kept in their respective agar and stored at 4 °C during the determination. The microorganisms were deposited at the company Water supply and sewerage, Podgorica, Montenegro. After preparing the microtiter plates, they were incubated at 37 °C for 24 h and then resazurin (Sigma Aldrich, St. Louis, MO, USA) was added. The results are determined 4 h later.

### 2.6. Determination of Antioxidant Activity of Pine Extracts

#### 2.6.1. Free Radical Scavenging Activity—DPPH Assay

The conventional DPPH method described by Akpinar et al. (2016) [39] was applied, in which antioxidant activity is expressed as µmol Trolox equivalents per gram of sample. This approach is consistent with other studies on *Pinus* species [40,41,42] where results are likewise reported as Trolox equivalents rather than IC_50_ values. Reagents included methanol (99%), DPPH (0.5 mM; prepared by dissolving 0.02 g in 100 mL methanol and stored in the dark for 24 h), and Trolox (6-hydroxy-2,5,6,7,8-tetramethylchroman-2-carboxylic acid) as the reference antioxidant.

For the assay, 1 mL of extract, 1 mL methanol, and 0.5 mL DPPH solution were mixed in test tubes and incubated in the dark for 30 min. Absorbance was measured at 517 nm against a blank containing only the extraction solvent. Background absorbance of the extract prior to DPPH addition was subtracted. The percentage of DPPH inhibition was calculated as:

$$\%\mathrm{Inhibition}\;=\;\frac{Acontrol-Asample}{Acontrol}\times 100$$

- Acontrol—absorbance of the control (DPPH solution without extract)
- Asample—absorbance of the sample after reaction with the extract

A Trolox calibration curve was constructed by preparing serial dilutions (0, 25, 50, 100, 200, 300 µL) (Table 2) of 0.5 mM Trolox solution in 2 mL methanol with 400 µL DPPH. After 30 min, absorbances were measured, and the calibration curve (y = −0.0027x + 0.9069, R^2^ = 0.8893; y = absorbance, x = Trolox concentration in mg/mL) was used to express antioxidant activity as mg Trolox equivalents (mg TE) per 100 g of dry matter. All measurements were performed in triplicate, and data analysis was conducted using Microsoft Excel.

#### 2.6.2. Ferric Reducing Antioxidant Power (FRAP) Assay

The determination of antioxidant activity using the FRAP (Ferric Reducing Antioxidant Power) method was carried out according to Benzie and Strain (1996) [43] with some modifications [44]. The principle of evaluating the potential antioxidant activity of the analyzed sample is based on the redox reaction between the extract—the electron donor—and the Fe^3+^ ion—the electron acceptor, whereby the Fe^3+^ ion is reduced to Fe^2+^ ion. The reduction is monitored spectrophotometrically by turning the original yellow color of the Fe^3+^ TPTZ complex solution into a blue colored Fe^2+^ TPTZ complex.

The results are converted using the formula and expressed as µmol/L FRAP:

$$\frac{absorbance\;of\;the\;tested\;sample\;at\;a\;wavelenght\;of\;593\;nm}{absorbance\;of\;the\;standard\;solution\;at\;a\;wavelength\;of\;593\;nm}\times {Fe}^{2+}\;concentration\;of\;the\;standard\;(µmol/L)$$

### 2.7. Determination of the Effect of Pinus Extracts on the Cell Cycle of HeLa Cells

The effects of the tested compounds on the distribution of cell cycle phases were estimated by propidium iodide (PI) staining of the cells and subsequent FACS analysis as described in Sova M et al. (2013) [45]. In brief, after the indicated treatment, 0.5 × 10^6^ cells per sample were harvested, washed with PBS and fixed with 70% ethanol. After overnight incubation at 4 °C, cells were washed in PBS and resuspended in 500 µL staining buffer (100 mM Tris, pH 7.4, 150 mM NaCl, 1 mM CaCl_2_, 0.5 mM MgCl_2_, 0.1% Nonidet P-40) containing 3 µM propidium iodide (PI) and 100 µg/mL RNase A and incubated at 37 °C for 30 min. The cells were zanalyzed with the FACS Calibur (Becton Dickinson, San Jose, CA, USA).

#### Citotoxic Activity of Pinus Extracts on Cancer Cell Lines

For the purpose of biological activity evaluation of tested samples, MTT test was performed.

The cell lines of human cervical adenocarcinoma (HeLa), human malignant melanoma (Fem-x) and normal, human lung fibroblast (MRC-5) were maintained in complete nutrient medium RPMI-1640 at 37 °C in humidified atmosphere with 5% CO_2_. All cell lines were obtained from American Type Culture Collection (Manassas, VA, USA). For all of the cells used, the nutrient medium was RPMI 1640 (Sigma, USA) supplemented to final concentration with L-glutamine (3 mM), streptomycin (100 mg/mL), and penicillin (100 IU/mL), fetal bovine serum (10%; FBS; 56 °C heat inactivated due to inactivation of cholinesterases and system complement and HEPES (25 mM)), adjusted to pH 7.2 (bicarbonate solution). For cell survival de-terminations, the 3-(4,5-dimethylthiazol-2-yl)-2,5-diphenyl tetrazolium bromide—MTT (Sigma, USA) was dissolved in phosphate-buffered saline, pH 7.2 (5 mg/mL).

The HeLa (2 × 10^3^ cells/100 μL per well), LS 174 (7 × 10^3^ cells/100 μL per well), Fem-x (2 × 10^3^ cells/100 μL per well), and MRC-5 cells (5 × 10^3^ cells/100 μL per well) were seeded into 96-well microtiter plates. Twenty hours later, after cell adherence, five different concentrations of the test compounds were added to the wells. The final test compound concentrations were from 2 to 33 cells and culture medium were used as controls.

All of the experiments were carried out in triplicate. Nutrient medium with the corresponding concentrations of the test compounds, but void of cells, was used as the blank.

Cell survival was determined by the MTT test according to the method of Mosmann [46], and at 48 h after the compound additions. Briefly, 20 μL MTT solution (5 mg/mL in the PBS) was added to each well. The samples were incubated for additional 4 h at 37 °C, in 5% CO_2_ and humidified atmosphere. Then, 100 μL 10% sodium dodecylsulfate—SDS (Sigma, USA) was added to each of the wells, and the absorbance of the cell medium from each well was measured at 570 nm the next day. Measurements were performed using Multiskan™ FC Microplate Photometer (Thermo Scientific, Waltham, MA, USA) [44].

## 3. Results and Discussion

### 3.1. The Total Phenolic, Flavonoid and Tannins Contents of Pinus sp. Plant Extracts

Phenolic compounds are among the most important classes of secondary metabolites, known to function as primary antioxidants and free radical scavengers [47]. Therefore, quantifying their content in the selected *Pinus* species is essential. Polyphenols play a crucial role in plant defense mechanisms against pathogens and harmful ultraviolet (UV) radiation [48]. Flavonoids, phenols, and tannins contribute to the neutralization of free radicals, thereby reducing or preventing cellular and tissue damage. The present study found moderate levels of these compounds across various *Pinus* species, suggesting a possible role in reducing oxidative stress. The results of total phenolic content (TPC), total flavonoid content (TFC), and total tannin content (TTC) are presented in Table 3.

The observations from Table 3. show that the total phenolic content (TPC) is highest in the extracts obtained by ultrasonic extraction and that it differs depending on the *Pinus* species. The total phenolic content was particularly highest in the extract of *P. sylvestris* (3.438 mg/g GAE), followed by the extract of *P. heldreichii* (2.732 mg/g GAE) and *P. pinea* (2.423 mg/g GAE) (Table 3). Ultrasonic-assisted extraction (UAE) was found to be the most efficient method, yielding the highest levels of total phenols and tannins in the majority of cases. These findings confirm the superior capacity of UAE for the isolation of polyphenolic compounds. In contrast, digestion (D) demonstrated particular effectiveness in the extraction of flavonoids, with *P. pinea* exhibiting the most pronounced response. Classical maceration (CM), when compared with both digestion and ultrasonic-assisted extraction, consistently resulted in lower yields across all evaluated compound classes, highlighting its lower efficiency. Among the investigated pine species, *Pinus pinea* emerged as the richest source of flavonoids and tannins, whereas *Pinus sylvestris* was distinguished by the highest phenolic content.

Comparison with literature data (Table 4), although direct quantitative evaluation is not possible due to differences in units and extraction protocols, allows the identification of general trends. It is confirmed that *Pinus sylvestris* and *Pinus heldreichii* generally contain high levels of phenolic compounds, whereas *Pinus pinea* is particularly rich in flavonoids and tannins. It is evident that the extraction method and the plant organ used significantly influence the final values, highlighting the importance of carefully selecting the extraction technique in studies of bioactive compounds.

The total flavonoid content (TFC) was particularly the highest in the extract of *P. pinea* (1.213 mg/g QE) obtained by digestion (D), followed by the extracts of *P. heldreichii* (1.074 mg/g QE) and *P. halepensis* (1.074 mg/g QE), both obtained by ultrasonic extraction (Table 3).

The tannin content ranged from 4.372 to 8.214 mg/g GAE, depending on the *Pinus* species and extraction method. The highest tannin content was found in the *P. pinea* sample and amounted to 8.214 mg/g GAE, while it was significantly lower in the *P. nigra* sample and ranged from 4.372 to 4.732 mg/g GAE (Table 3).

The Pearson correlation analysis between total phenolic content (TPC), total flavonoid content (TFC), and total tannin content (TTC) in extracts of different *Pinus* species showed that the interrelationship among these bioactive compounds in the examined samples was not significant. Total phenols and flavonoids exhibited a very weak negative correlation (−0.074), suggesting that an increase in phenolic content does not necessarily correspond to an increase in flavonoids in these extracts. The correlation between total phenolic content and tannins was slightly positive (0.195), indicating a tendency for extracts with higher phenolic content to also contain somewhat higher levels of tannins. Similarly, flavonoids and tannins showed a very weak positive correlation (0.074).

Analysis of TPC, TFC, and TTC values (Table 3) clearly indicates the possibility of grouping different pine species based on their bioactive profiles. Specifically, *P. halepensis* and *P. nigra* exhibit similar concentrations of phenols, flavonoids, and tannins across all applied extraction methods, placing them in the same or closely related group. In contrast, *P. pinea* and *P. sylvestris* stand out due to significantly higher tannin and phenol content, particularly in the ultrasound-assisted extraction, suggesting potentially greater bioactivity of their extracts. These differences highlight the importance of both species and extraction method in optimizing extracts to achieve maximal bioactive effect.

Many researchers attribute the variability in total phenolic content (TPC) within the same *Pinus* species to differences in bioclimatic conditions at the growth sites, particularly variations in rainfall, soil composition, and temperature [49,50]. Other studies suggest that genetic variability plays a significant role, as demonstrated in research on black pines (*Pinus nigra*) [51]. Additionally, some authors report a positive correlation between environmental stress and the accumulation of phenolic compounds, proposing that these compounds may serve as biomarkers for assessing plant exposure to stress [52].

### 3.2. Antioxidant Activity of Various Needle Extracts of Pinus Taxa Using DPPH and FRAP

The antioxidant activity of the dry needle extracts of *Pinus* taxa was studied using two different assays, namely the 2,2-diphenyl-1-picrylhydrazyl radical scavenging (DPPH) and the ferricyanide reducing power assay (FRAP). The results of antioxidant activity are shown in Table 5. Regarding the antioxidant activity quantified by DPPH in each of the *Pinus* species studied, the lowest value was obtained for *P. heldreichii* (239.96 mg Trolox equivalent (TE) per 100 g dw, followed by *P. pinea* (242.93 mgTE/100 g dw). The species with the highest values were *P. halepensis* with 288.85 mgTE/100 g dw and *P. nigra* with 285.52 mg TE/100 g dw (Table 5). In *P. sylvestris* extracts, the recorded DPPH values ranged from 249.22 to 278.48 mg TE/100 g dw), Regarding the antioxidant activity quantified with the FRAP, the species with the highest values were *P. pinea* with 430.89 μmol/L and with the lowest antioxidant activity *P. halepensis* (56.27 μmol Fe^2+^/L). The results indicate that different mechanisms of antioxidant activity are present among In general, there are no large variations in DPPH activity of dry needles of *Pinus* taxa; however, variations in FRAP activity are visible, so that the values for the studied species ranged from 56.27 to 430.89 μmol Fe^2+^/L (Table 5). It must be emphasized that this categorization based on the DPPH and FRAP assay may serve as a useful tool for the selection of *Pinus* species with higher or lower antioxidant activity. Considering this aspect, the difference in quantitative antioxidant activity between species usually varies depending on the method.

**Figure 1:**
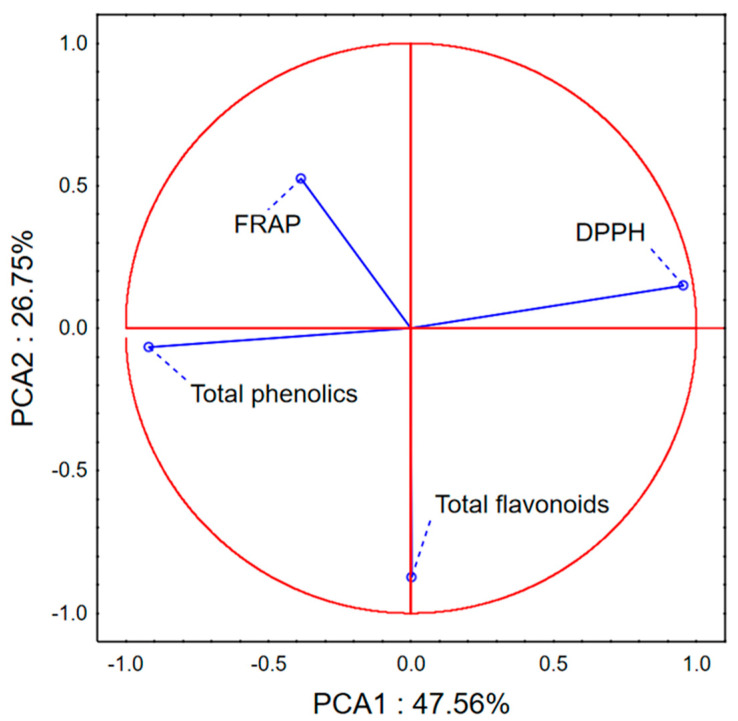
Data relevant to statistical analysis—PCA loadings.

**Figure 2:**
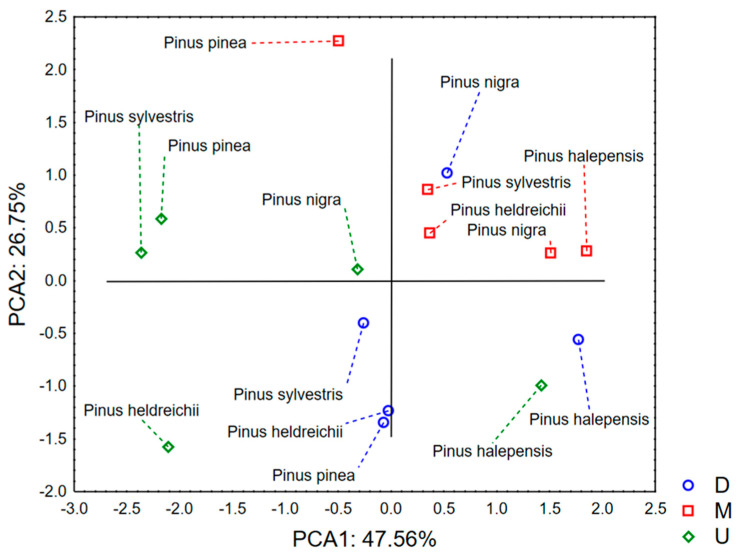
The position of the examined samples in the space defined by the first two factor axes.

The application of principal component analysis to the data set describing the amounts of total phenols and flavonoids in the studied samples, as well as their antioxidant potential assessed by DPPH radical scavenging assay and FRAP, shows that the first two principal components describe about 74% of the variability of the data set (Figure 1 and Figure 2). Variables analyzed:

- Amounts of total phenols and total flavonoids
- Antioxidant potential estimated in the FRAP and DPPH assay.

In the first principal component (PCA1), most of the variability among the samples correlates with the quantified levels of total phenols and the ability of the extracts to scavenge DPPH radicals (Figure 1). In contrast, variability along the second principal component (PCA2) is primarily associated with the total flavonoid content. The distribution of samples in the space defined by PCA1 and PCA2 reveals a clustering of *Pinus* sp. extracts obtained by conventional maceration (CM) in the positive region of PCA1, reflecting their higher DPPH radical scavenging activity (Figure 2). Conversely, extracts obtained by ultrasonic extraction (U) are mostly located in the negative region of PCA1, suggesting a higher iron-reducing antioxidant capacity but lower DPPH scavenging potential. Notably, ultrasonic extraction also enhances the overall yield of extracted polyphenols. With respect to PCA2, extracts obtained by digestion (D) tend to exhibit higher total flavonoid content, although this appears to be species-dependent. Regardless of the extraction method, *Pinus halepensis* extracts consistently group in the positive region of PCA1, indicating strong DPPH radical scavenging capacity but weak iron (III) reduction potential. In contrast, *Pinus nigra* extracts display moderate antioxidant activity in both assays employed.

### 3.3. Quantification of Secondary Metabolites in Extracts of Pinus *sp*.

The identification and quantification of phenolic acids was determined in the needle extracts of *Pinus* taxa by HPLC analysis. HPLC quantification of the secondary metabolites revealed that the values of phenolic acids varied depending on the type and method of extraction (Table 6). The highest values for trans-cinnamic acid were determined in *P. halepensis* (218.9 µg/g) using the UAE method. Trans-cinnamic acid detected in the present study ranged from 38.3 to 218.9 µg/g, depending on the pine species and extraction method. Although this compound exhibits well-documented biological activities, including antioxidant, antimicrobial [53], anticancer [54], neuroprotective [55], anti-inflammatory, and antidiabetic effects, the concentrations reported in these studies are substantially higher than those found in our extracts. For example, significant anticancer activity of pure cinnamic acid typically occurs at 1480–5920 µg/g, which is one to two orders of magnitude above the levels quantified here. The presence of cinnamic acid in the range of 40–220 µg/g in *Pinus* extracts is still relevant, as this compound is a known contributor to the phenolic profile of conifers and may enhance the biological properties of the extracts through synergistic interactions with other phenolic acids and flavonoids.

Caffeic acid was abundant in *P. sylvestris* (935.6 µg/g), and p-coumaric acid was also high (158.1 µg/g) when using the CM extraction method. The dominant components detected in the extracts of *Pinus* species by HPLC analysis were ferulic acid (5.9–1563.5 µg/g), rutin (58.5–2254.7 µg/g) and quercitrin (68.5–1537.9 µg/g). The highest levels of ferulic acid were found in *P. sylvestris* (1563.5 µg/g) and *P. halepensis* (1552.7 µg/g). No significant differences were found in the ferulic acid values in the extracts of *Pinus* sp. obtained by different extraction methods. The highest rutin concentration was observed in *P. sylvestris* extracts (2254.7 µg/g), while no rutin was detected in *P. heldreichii* extracts. The highest levels of quercitrin (1537.9 µg/g) were found in *P. pinea* extracts, with significantly lower levels in *P. heldreichii* extracts (68.5 µg/g). Quercetin was only detected in *P. pinea* extracts, with concentrations ranging from 52.9 to 87.6 µg/g. Quercetin has also been identified in other pine species [56], specifically *P. sylvestris* from Poland, where concentrations ranged from 155.76 to 243.74 μg/g dw [48]. Rosmarinic acid was not detected in any extracts of *Pinus* taxa. Gallic acid was present in *P. halepensis* extracts (193.8–232.8 µg/g) and in significantly lower concentrations in *P. pinea* extracts (145.9–170.2 µg/g). Gallic acid was not detected in *P. sylvestris* extracts from Montenegro, but was identified in *P. sylvestris* from Poland, with concentrations ranging from 134.57 to 184.54 μg/g dw [48]. Additionally, *P. sylvestris* extracts from Montenegro exhibited substantially higher rutin levels compared to *P. sylvestris* extracts from Poland. In contrast, *P. sylvestris* extracts from Poland contained significantly higher concentrations of t-cinnamic acid, caffeic acid, p-coumaric acid, chlorogenic acid, and ferulic acid compared to extracts from all pine species collected in Montenegro (Table 6).

The concentrations of phenolic acids and flavonoids in *Pinus* needle extracts are largely consistent with previously reported values in the literature. For instance, in *P. sylvestris* UAE extracts, rutin was quantified at 2254.7 µg/g and caffeic acid at 901.0 µg/g, which are higher than those reported in *P. sylvestris* shoots (~6.3 µg/g rutin, ~15,020 µg/g caffeic acid) [53,57]. Similarly, in *P. halepensis* digestion (D) extracts, trans cinnamic acid reached 73.3 µg/g, ferulic acid 1464 µg/g, and chlorogenic acid 84 µg/g, which are within the range of phenolic acids previously reported in needles and bark (~1110 µg/g trans cinnamic acid, 20,890 µg/g ferulic acid, 5180 µg/g chlorogenic acid) [55,56]. Results in this study demonstrate that the extraction method strongly affects the concentration of bioactive compounds: Ultrasound-assisted extraction (UAE) has been demonstrated to yield higher concentrations of phenolic and other bioactive compounds from *Pinus* spp. than classical extraction methods such as maceration or Soxhlet extraction [57,58,59]. Overall, these findings highlight the importance of both extraction method and plant species in determining the phenolic profile of *Pinus* extracts and confirm their potential as sources of bioactive polyphenols.

**Figure 3:**
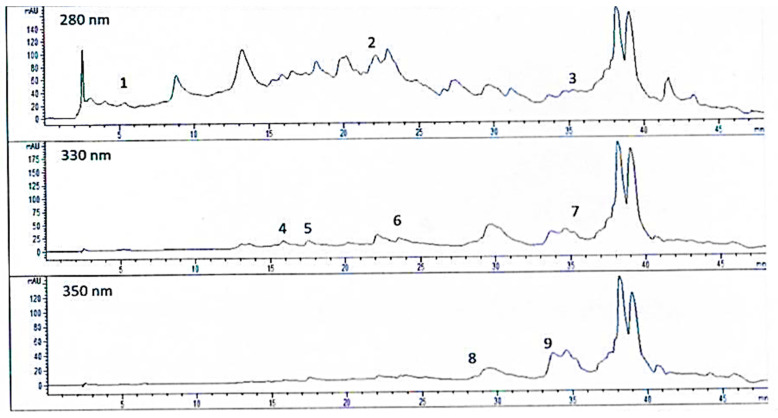
HPLC-DAD chromatogram. Detected compounds: **1**. gallic acid; **2**. caffeic acid; **3**. trans-cinnamic acid; **4**. p-coumaric acid; **5**. quercetin; **6**. chlorogenic acid; **7**. ferulic acid; **8**. rutin; **9**. quercitrin.

The findings reveal pronounced interspecific differences in the phenolic composition of the analyzed pine species. *Pinus halepensis* was characterized by a notably high content of ferulic acid, accompanied by considerable amounts of rutin and quercitrin. In *Pinus nigra*, chlorogenic acid and rutin were the predominant constituents, although the overall concentrations remained moderate compared to the other species. *Pinus heldreichii* was the species with the lowest overall phenolic content within the group, yet it was characterized by a relatively high level of caffeic acid. The highest phenolic abundance was observed in *Pinus sylvestris*, primarily attributable to its substantial levels of ferulic acid, rutin, and caffeic acid, highlighting its strong antioxidant potential. Finally, *Pinus pinea* exhibited the most diverse phenolic profile, being the only species in which quercetin was detected, alongside elevated concentrations of quercitrin and chlorogenic acid, underscoring its unique phytochemical composition. HPLC chromatograms of the phenolic compounds in the five *Pinus* taxa are shown in Figure 3.

Retention time (tR) and *m*/*z* values were not determined in this study because only HPLC–DAD analysis was performed; identification was based on comparison with standards and literature data.

### 3.4. Antimicrobial Potential

Antibacterial activity was assessed by determining the Minimum Inhibitory Concentration (MIC) using the broth dilution method. As shown in Table 7, the MIC values of *Pinus* species extracts against the tested bacterial strains ranged from 19 to 66 mg/mL, which are higher than the previously reported MIC values for *Pinus* taxa (3.8–15 mg/mL) [60]. The positive control against the tested bacterial strains (ATCC) is presented in Table 7. The extract of *Pinus pinea* exhibited an MIC of 19 mg/mL against *Enterococcus faecium*, while *Pinus sylvestris* showed activity against *Bacillus spizizenii*. Among the tested samples, *P. sylvestris* and *P. pinea* demonstrated the strongest antimicrobial activity against Gram-positive bacterial strains, suggesting higher concentrations of active phenolic compounds. Regarding Gram-negative bacteria, *P. halepensis* and *P. pinea* showed notable activity, with an MIC of 29 mg/mL against *Pseudomonas aeruginosa* and *Salmonella enteritidis* (D). The demonstrated antimicrobial activity of pine needle extracts against both Gram-positive and Gram-negative bacteria suggests the presence of bioactive compounds with antibacterial potential, which may be further explored for development as natural antibacterial agents.

The present study highlights the antimicrobial potential of extracts from various pine species against Gram-positive bacteria, revealing notable interspecific differences in efficacy. Among the tested species, *Pinus sylvestris* demonstrated superior inhibitory activity against *Bacillus spizizenii* and *Staphylococcus aureus*, while *Pinus pinea* exhibited pronounced selective activity against *Enterococcus faecium*. In comparison, extracts of *Pinus halepensis*, *Pinus nigra*, and *Pinus heldreichii* displayed moderate antimicrobial effects across the majority of strains, suggesting a consistent yet comparatively lower level of bioactivity. These findings underscore the species-specific nature of antimicrobial activity in pine extracts and their potential as sources of bioactive compounds for further investigation.

The present study demonstrates that extracts from different pine species exhibit inhibitory activity against Gram-negative bacteria, albeit with notable interspecific variability. *Pinus pinea* was particularly effective against *Pseudomonas aeruginosa*, displaying the lowest MIC value (29 mg/mL), indicative of a pronounced antimicrobial potential. *Pinus halepensis* and *Pinus nigra* showed relatively consistent moderate activity across the majority of tested strains, including *Proteus mirabilis*, *Escherichia coli*, and *Klebsiella* spp. In contrast, extracts of *Pinus heldreichii* and *Pinus sylvestris* generally exhibited moderate antimicrobial effects, with comparatively reduced efficacy against certain strains such as *Klebsiella aerogenes*, *Proteus mirabilis*, and *Salmonella enteritidis*. Furthermore, in studies of essential oils (EOs) from Balkan pine species, including *Pinus halepensis* and *Pinus sylvestris*, MIC values against bacteria ranged from 100 to 1000 µg/mL (0.1–1.0 mg/mL), with cones and needles often showing the strongest activity [61]. Compared to our data, antimicrobial activity derived from essential oils is markedly stronger, supporting the well-established notion that lipophilic, volatile compounds (terpenes, terpenoids) in oils are more effective antibacterial agents than polar, phenol-rich extracts. Studies of polar bark extracts from *Pinus pinaster* and *Pinus pinea* have shown bactericidal effects against Gram-positive *Staphylococcus aureus* at concentrations ranging from 6.25 to 25 mg/mL [42]. In that study, *P. pinea* bark extracts were effective even at 3.13 mg/mL, which is substantially lower than most of our MIC values for needle extracts. This suggests that differences in the plant part used (bark versus needles) and the extraction method (plant-specific polar bark extracts versus whole-needle or crude extracts) significantly influence antimicrobial potency.

### 3.5. Determination of the Effect of Pinus Extracts on the Cell Cycle

The distribution of cell cycle phases was studied by analyzing the DNA content using flow cytometry [45].

**Figure 4:**
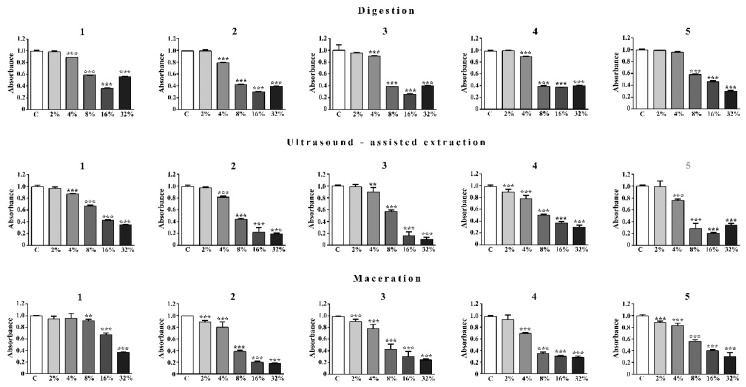
Distribution of HeLa cells across the G0/G1, S, and G2/M phases in the control group and after treatment with extracts obtained using different extraction methods. Statistical significance relative to the corresponding control phase is indicated as follows: *G0/G1 phase:* ** *p* < 0.005, *** *p* < 0.001.

Regarding the percentage of HeLa cells in the sub-Go/G1 phase of the cells cycle, i.e., cells with a lower DNA content than diploid and presumably apoptotic cells [62], treatment with all tested substances at concentrations of 8%, 16% and 32% resulted in an increase in cells in this phase compared to untreated cells (Figure 4).

Sample 1 and 4 obtained by digestion, sample 3 obtained by ultrasonic extraction and sample 3 obtained by maceration affected the S phase of the cell cycle and led to a significant increase in viable cells in this phase at all three concentrations tested. Sample 2 obtained by maceration showed the same effect at the lowest (8%) and highest (33%) concentrations, and substance 2 obtained by ultrasonic extraction at a concentration of 16%. Sample 2 obtained by digestion affected the S phase by causing an accumulation of cells in this phase at the lowest and at the highest concentration applied (33%). Samples 3 and 5 obtained by digestion, samples 1, 4 and 5 obtained by ultrasonic extraction and samples 1 and 4 obtained by maceration affected different phases of the cell cycle, the effect depending on the concentration applied. Sample 3 obtained by digestion induced not only cell cycle arrest in the S phase at all concentrations tested, but also an accumulation of viable cells in the G2/M phase at the lowest concentration. Sample 5, obtained by digestion, causes an increase in the proportion of viable cells in the S phase at the lowest concentration, in the S and G2/M phases at the medium concentration and in the G0/G1 phase at the highest concentration. Sample 1, obtained by ultrasonic extraction, resulted in a significant increase in viable cells in the GoG1 phase at the lowest concentration tested (8%) and in the S phase at the highest concentration. Sample 4, obtained by ultrasonic extraction, led to an accumulation of cells in the G0/G1 phase at the lowest concentration and in the S phase at higher concentrations. Sample 5, extracted by ultrasound, led to an accumulation of cells in the S phase at the lowest concentration and in the G0/G1 phase at the medium concentration (16%).

The distribution of cell cycle phases of viable HeLa cells treated with the tested samples varied greatly depending on the concentration used and the extraction method. Compounds extracted from the needles of five *Pinus* species by digestion, administered in at least one of the tested concentrations, induced an accumulation of cells in S phase, compared to untreated cells. In addition, compound **3** at a concentration of 8% and compound **5** at a concentration of 16% induced cell cycle arrest in the G2/M phase, i.e., in the mitotic phase. The compounds obtained from the needles of five *Pinus* species by ultrasound–assisted extraction, administered in at least one of the tested concentrations, also led to an increase in the fraction of treated cells in the S phase compared to the untreated cells. Compounds **1** and **4** at a concentration of 8% and compound **5** at a concentration of 16% caused an increase in the fraction of treated cells in the G0/G1 phase compared to the untreated cells. When the compounds obtained from the needles of five *Pinus* species were extracted by maceration, the tested compounds induced an accumulation of treated cells in the S phase in at least one of the applied concentrations. The highest concentration tested (32%) of compound **1** and 16% and 32% of compound **4** induced the accumulation of cells in the Go/G1 phase of the cell cycle.

The cell-cycle alterations observed in this study are broadly consistent with previously reported effects of pine-derived polyphenols and related phenolic extracts. Our findings show that the pine needle extract induces G1 and G2/M cell-cycle arrest, as well as an increase in the sub-G0/G1 fraction at higher concentrations, indicating apoptosis. These effects are consistent with earlier reports demonstrating that pine needle–derived extracts and constituents can modulate cell-cycle progression in various cancer cell lines. For example, pine needle oil was shown to induce G2/M arrest via ATM-mediated DNA damage response in hepatoma cells [63], hexane extracts triggered G1 arrest with p27^KIP1 upregulation in gastric cancer cells [64], tannin-rich pine bark extracts induced G2/M arrest and apoptosis in HeLa and U2OS cells [65] and α-pinene caused G2/M arrest in hepatoma cells [66]. These parallels confirm that our observations are aligned with established biological responses to pine-derived extracts. Where the direction of cell-cycle arrest depends strongly on the phenolic composition of the extract and, consequently, on the extraction technique used.

### 3.6. Determination of Survival

For the purpose of biological activity evaluation of tested samples, MTT test was performed. The cell lines of human cervical adenocarcinoma (HeLa), human malignant melanoma (Fem-x) and normal, human lung fibroblast (MRC-5) were maintained in complete nutrient medium RPMI-1640 at 37 °C in humidified atmosphere with 5% CO2. All cell lines were obtained from American Type Culture Collection (Manassas, VA, USA). For all of the cells used, the nutrient medium was RPMI 1640 (Sigma, Ronkonkoma, NY, USA) supplemented to final concentration with L-glutamine (3 mM), streptomycin (100 mg/mL), and penicillin (100 IU/mL), fetal bovine serum (10%; FBS; 56 °C heat inactivated due to inactivation of cholinesterases and system complement and HEPES (25 mM)), adjusted to pH 7.2 (bicarbonate solution). For cell survival determinations, the 3-(4,5-dimethylthiazol-2-yl)-2,5-diphenyl tetrazolium bromide—MTT (Sigma, USA) was dissolved in phosphate-buffered saline, pH 7.2 (5 mg/mL). The HeLa (2 × 10^3^ cells/100 μL per well), LS 174 (7 × 10^3^ cells/100 μL per well), Fem-x (2 × 10^3^ cells/100 μL per well) and MRC-5 cells were seeded (5 × 10^3^ cells/100 μL per well) into 96-well microtiter plates, and 20 h later, after cell adherence, five different concentrations of the test compounds were added to the wells. The final test compound concentrations were from 12.5 μg/mL to 200 μg/mL. Only nutrient medium was added to the cells in the control wells. All of the experiments were carried out in triplicate. Nutrient medium with the corresponding concentrations of the test compounds, but void of cells, was used as the blank. Cell survival was determined by the MTT test according to the method of Mosmann [46], and at 48 h after the compound additions.

For the purpose of analyzing the results, we assigned numbers to the samples (Table 8).

**Figure 5:**
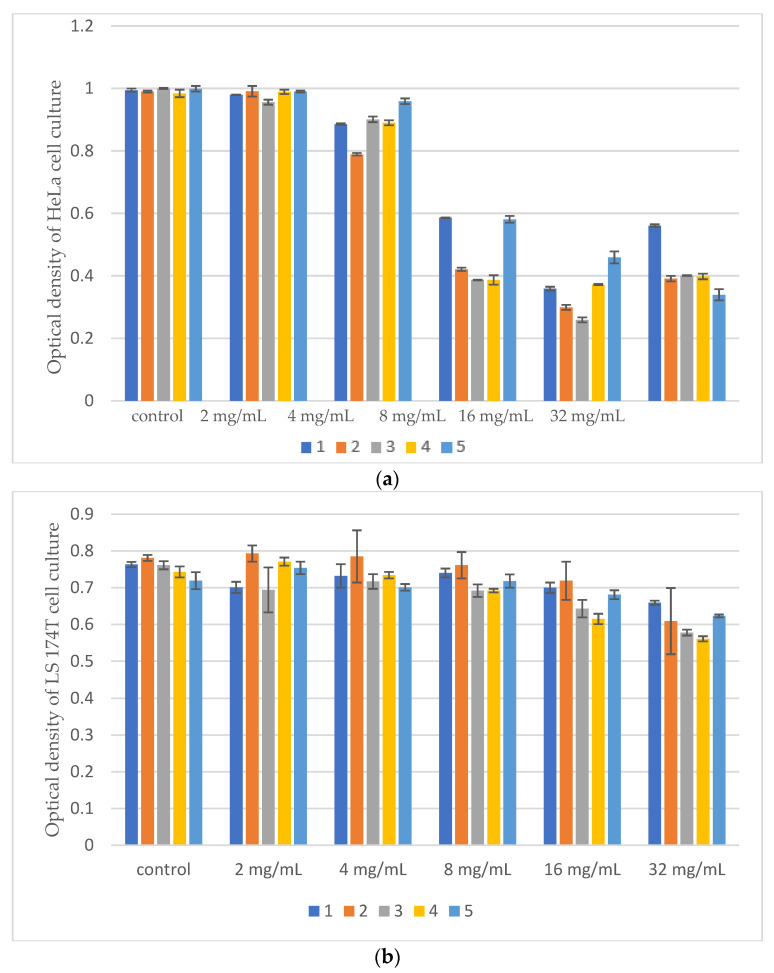

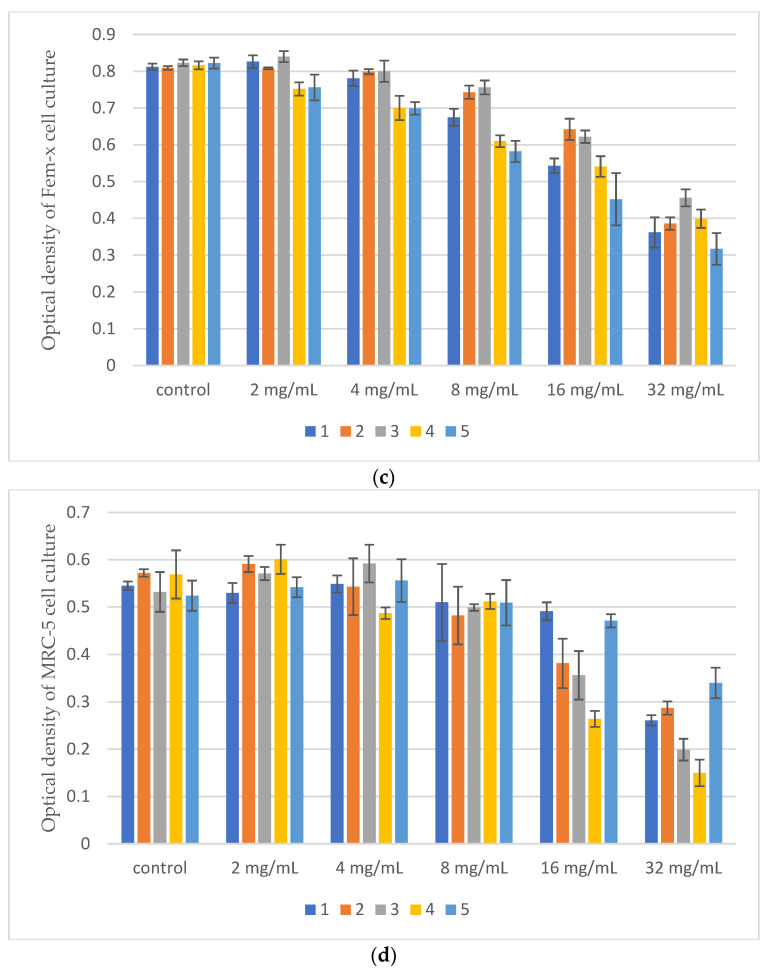
(**a**) Optical density of Hela cell culture after treatment with different concentrations of samples obtained by digestion. (**b**) Optical density of LS 174T cell culture after treatment with different concentrations of samples obtained by digestion. (**c**) Optical density of Fem-x cell culture after treatment with different concentrations of samples obtained by digestion. (**d**) Optical density of MRC-5 cell culture after treatment with different concentrations of samples obtained by digestion.

**Figure 6:**
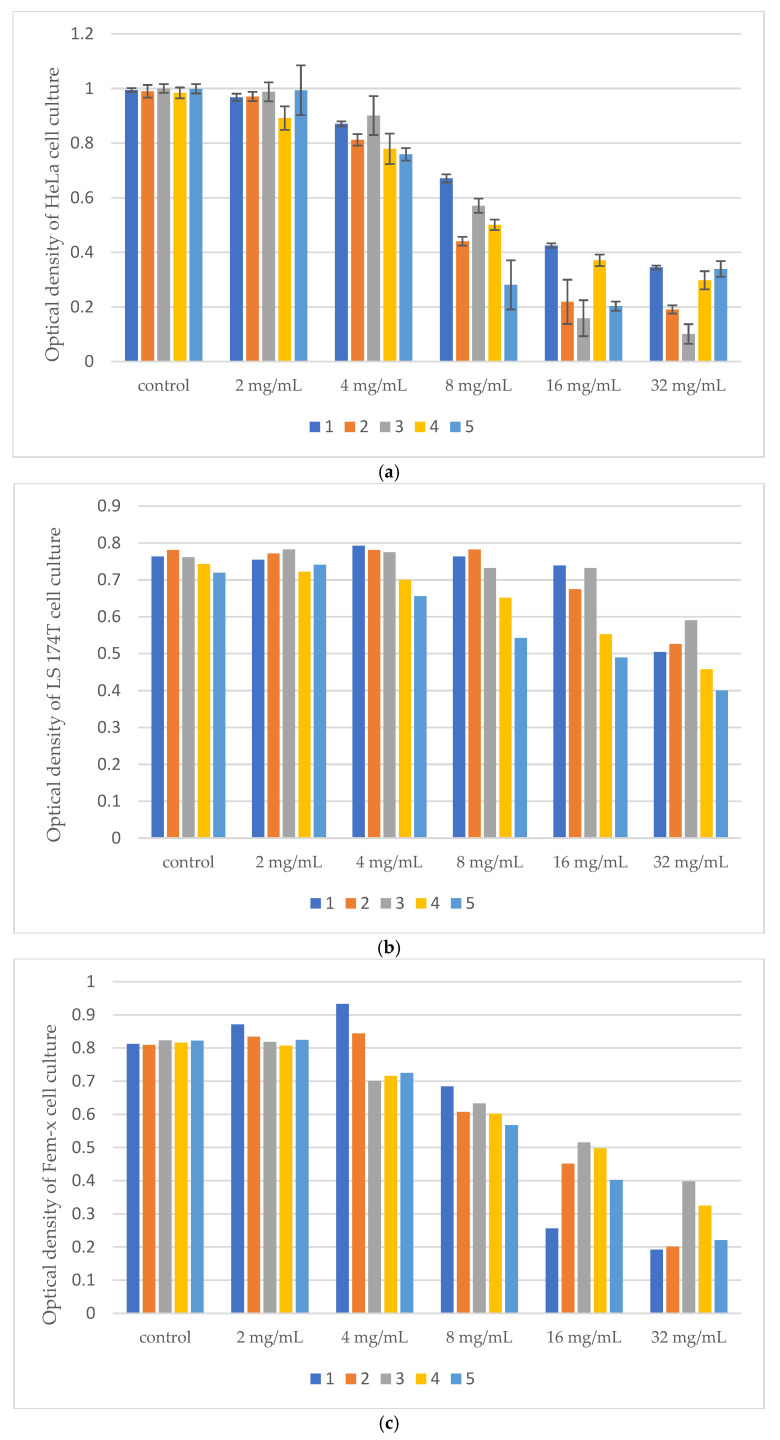

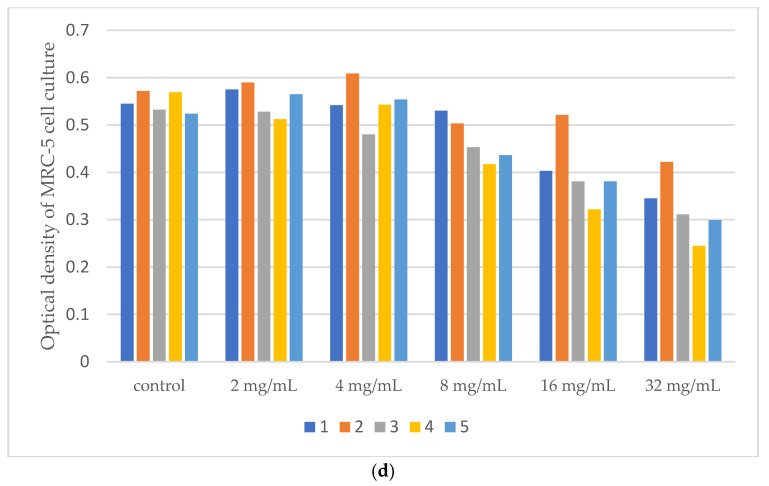
(**a**) Optical density of Hela cell culture after treatment with different concentrations of samples obtained by ultrasound-assisted extraction. (**b**) Optical density of LS 174T cell culture after treatment with different concentrations of samples obtained by ultrasound-assisted extraction. (**c**) Optical density of Fem-x cell culture after treatment with different concentrations of samples obtained by ultrasound-assisted extraction. (**d**) Optical density of MRC-5 cell culture after treatment with different concentrations of samples obtained by ultrasound-assisted extraction.

**Figure 7:**
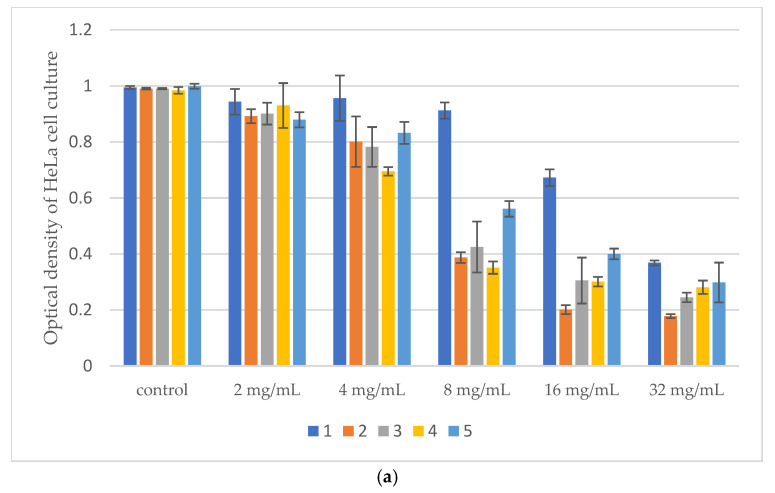

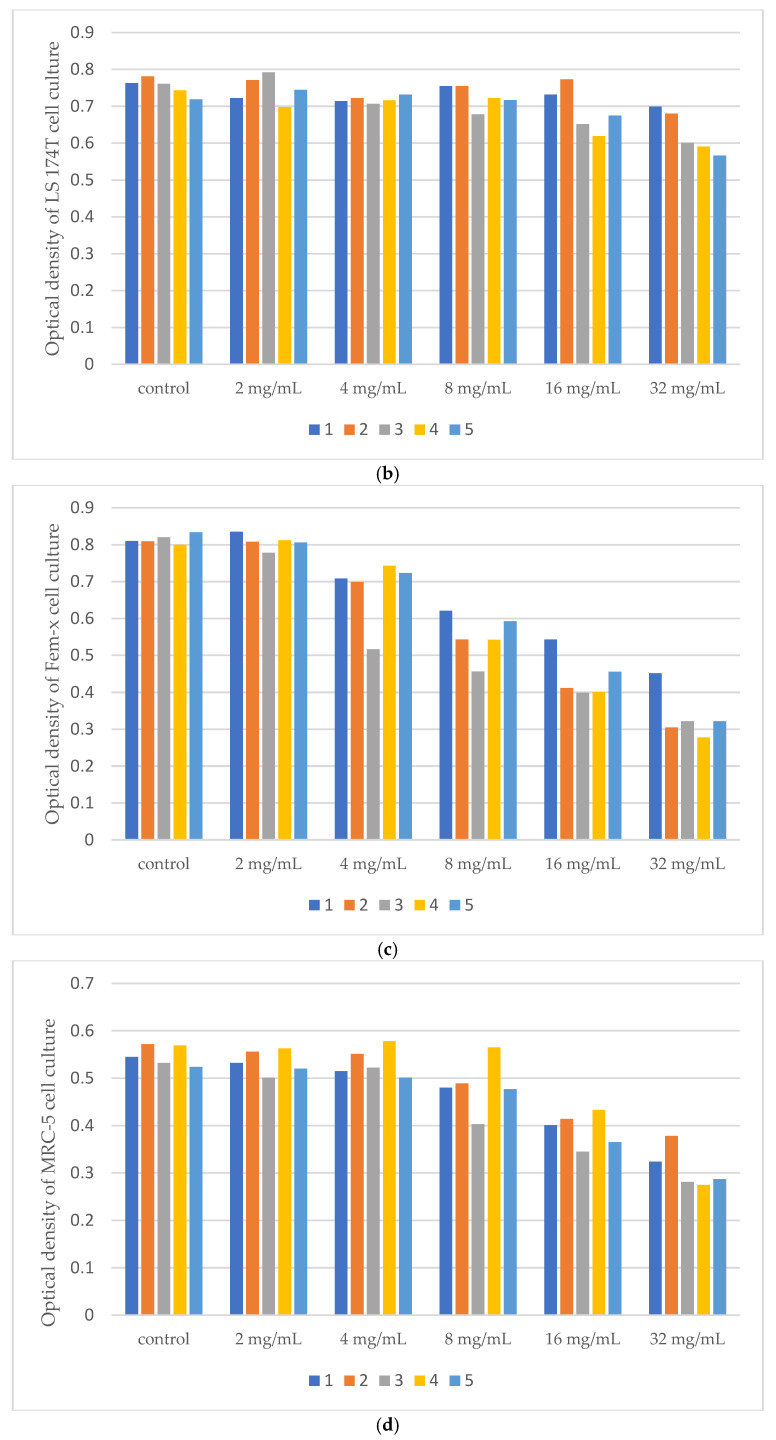
(**a**) Optical density of Hela cell culture after treatment with different concentrations of samples obtained by maceration. (**b**) Optical density of LS 174T cell culture after treatment with different concentrations of samples obtained by maceration. (**c**) Optical density of Fem-x cell culture after treatment with different concentrations of samples obtained by maceration. (**d**) Optical density of MRC-5 cell culture after treatment with different concentrations of samples obtained by maceration.

Results of Citotoxic Activity of *Pinus* Extracts on Cancer Cell Lines using MTT test was presented on Figure 5, Figure 6 and Figure 7.

All tested samples exhibited antiproliferative activity at concentrations of 8%, 16%, and 32% on HeLa cells while LS 174T cells were the least sensitive.

The IC_50_ values of the extracts obtained by the three extraction methods are presented in Table 9 providing a comparative overview of their cytotoxic activity across all tested cell lines.

IC_50_ values obtained from MTT assays are commonly used as quantitative indicators of cytotoxic potency in vitro [55]. The IC_50_ value represents the concentration of a compound that reduces cell viability by 50% compared to the untreated control. HeLa cells were the most sensitive to digestive and UAE extracts (IC_50_ = 6.57–12.05 mg/mL), while LS 174T cells were resistant (IC_50_ > 32 mg/mL) and Fem-x cells showed moderate sensitivity (IC_50_ ≈ 30–32 mg/mL). Normal MRC-5 cells were largely unaffected, indicating selective cytotoxicity of the extracts toward cancerous cells.

## 4. Conclusions

Species of the genus *Pinus* are recognized as a source of diverse secondary metabolites with antioxidant, anti-inflammatory, and antimicrobial properties. The results of this study indicate that the examined pine needle extracts contain a variety of bioactive compounds and show measurable antioxidant, antimicrobial, and dose-dependent antiproliferative effects. These findings provide initial insights into the chemical composition and biological activities of pine needles from Montenegro. The study lays a foundation for future investigations aimed at isolating individual constituents, clarifying their specific roles, and evaluating their safety and possible applications in areas such as pharmaceuticals, food products, and cosmetics. In this way, the research contributes to a better understanding of *Pinus* species and may support their sustainable use in developing natural value-added products.

## Figures and Tables

**Table 1:** Geographical position of the selected pine species, determined with the help of the GPS Altitude application, version 5.11 (278).

Species	*Pinus halepensis*	*Pinus nigra*	*Pinus heldreichii*	*Pinus sylvestris*	*Pinus pinea*
Latitude	42.265672	43.166594	42.551753	43.194750	42.392898
Longitude	19.107399	19.308508	18.523573	19.318036	18.597822
Elevation	19 m	1178 m	1146 m	1199 m	167 m

**Table 2:** Absorbance of Trolox standard solutions at 517 nm for calibration curve.

Trolox Volume (µL)	Absorbance
0	1.055
25	0.821
50	0.755
100	0.506
200	0.258
300	0.202

Note: All values are mean of triplicate measurements.

**Table 3:** Total phenolic (TPC), flavonoid (TFC), and Total tannin content (TTC) of five *Pinus* needle extracts expressed as mg/g dry plant material.

Pine Species	Extraction Type	TPC (mg/g GAE)	TFC (mg/g QE)	TTC (mg/g GAE)
*Pinus halepensis*	D	1.369 ± 0.005	0.754 ± 0.001	5.077 ± 0.254
	CM	0.894 ± 0.005	0.774 ± 0.281	5.849 ± 0.292
	UAE	1.369 ± 0.002	1.074 ± 0.09	4.936 ± 0.247
*Pinus nigra*	D	1.535 ± 0.003	0.459 ± 0.426	4.506 ± 0.225
	CM	1.335 ± 0.003	0.626 ± 0.174	4.732 ± 0.237
	UAE	2.044 ± 0.002	0.718 ± 0.044	4.372 ± 0.219
*Pinus heldreichii*	D	1.751 ± 0.003	0.872 ± 0.16	4.893 ± 0.245
	CM	1.563 ± 0.005	0.649 ± 0.206	5.937 ± 0.297
	UAE	2.732 ± 0.002	1.074 ± 0.045	4.869 ± 0.243
*Pinus sylvestris*	D	1.701 ± 0.003	0.939 ± 0.028	6.604 ± 33.02
	CM	1.763 ± 0.005	0.636 ± 0.09	6.619 ± 0.331
	UAE	3.438 ± 0.002	0.526 ± 0.029	6.448 ± 0.322
*Pinus pinea*	D	1.629 ± 0.002	1.213 ± 0.019	8.029 ± 0.402
	CM	1.713 ± 0.006	0.433 ± 0.089	7.314 ± 0.366
	UAE	2.423 ± 0.003	0.756 ± 0.143	8.214 ± 0.411

Values represent mean ± SD of three measurements (*n* = 3).

**Table 4:** Literature data on Total phenolic (TPC), flavonoid (TFC), and Total tannin content (TTC) of *Pinus* sp. needle extracts.

Species and Sample Type	TPC	TFC	TTC
*Pinus nigra* Methanolic needle extracts [26]	12.0–13.3 mg GAE/g	3.3 mg CE/g	N/A
*Pinus sylvestris* Methanolic needle extracts [26]	9.8–14.0 mg GAE/g	3.3–7.2 mg CE/g	N/A
*Pinus peuce* Methanolic needle extracts [26]	14.0 mg GAE/g	7.2 mg CE/g	N/A
*Pinus cembra* Hydromethanolic needle extract [27]	N/A	19.84 mg CE/g	N/A
*Pinus nigra* Ethanol needle extracts [28]	15.67–47.53 mg GAE/g	1.69–3.97 mg RE/g	10.44–36.81 mg CE/g
*Pinus heldreichii* Acidic methanol needle extracts [29]	31.65 mg g^−1^	11.20 mg g^−1^	N/A
*Pinus pinea* Bark extracts [30]	568 ± 12 mg GAE g^−1^	N/A	N/A
*Pinus halepensis* Methanol seed extracts [31]	12.69 ± 0.07–14.63 ± 0.05 mg GAE/g	N/A	N/A

Note: N/A means Not available.

**Table 5:** Relationship between the antioxidant activity of needle extracts of *Pinus* taxa using DPPH and FRAP.

Pine Species	Extraction Procedure	DPPH (mgTE/100 g of Dry Matter)	FRAP (μmol Fe^2+^/L)
*Pinus* *halepensis*	D	287.74 ± 0.11	56.27 ± 0.3
	CM	288.85 ± 0.13	228.13 ± 0.18
	UAE	288.85 ± 0.21	203.36 ± 0.64
*Pinus nigra*	D	271.44 ± 0.65	188.99 ± 0.35
	CM	285.52 ± 0.37	129.97 ± 0.18
	UAE	265.89 ± 0.21	217.13 ± 0.46
*Pinus heldreichii*	D	256.26 ± 0.4	73.09 ± 0.47
	CM	269.96 ± 0.21	217.13 ± 0.17
	UAE	239.96 ± 0.3	202.62 ± 0.35
*Pinus sylvestris*	DD	262.56 ± 0.21	275.84 ± 0.35
	CM	278.48 ± 0.21	279.20 ± 0.1
	UAE	249.22 ± 0.21	166.67 ± 0.46
*Pinus pinea*	D	265.52 ± 0.21	280.73 ± 0.31
	CM	267.37 ± 0.21	430.89 ± 0.35
	UAE	242.93 ± 0.21	389.60 ± 0.21

Values represent mean ± SD of three measurements (*n* = 3).

**Table 6:** Phenolic compound profile of the five *Pinus* taxa quantified through HPLC analysis.

	Phenolic Acids (µg/g)										
Pine Species	Extraction Type	Trans-Cinnamic Acid	Caffeic Acid	p-Coumaric Acid	Quercetin	Chlorogenic Acid	Rosmarinic Acid	Ferulic Acid	Gallic Acid	Rutin	Quercitrin
*Pinus halepensis*	D	73.3 ± 8.1	100.4 ± 5.0	3.4 ± 0.3	/	84.0 ± 4.2	/	1464.0 ± 87.8	226.1 ± 33.9	661.2 ± 52.9	815.5 ± 40.8
	CM	56.4 ± 6.2	101.8 ± 5.1	3.7 ± 0.4	/	61.8 ± 3.1	/	1092.1 ± 65.5	193.8 ± 29.1	589.7 ± 47.2	726.1 ± 36.3
	UAE	218.9 ± 24.1	85.2 ± 4.3	2.8 ± 0.3	/	66.9 ± 3.3	/	1552.7 ± 93.2	232.8 ± 34.9	622.1 ± 49.8	818.9 ± 40.9
*Pinus nigra*	D	131.7 ± 14.5	/	20.6 ± 2.1	/	211.7 ± 10.6	/	222.9 ± 13.4	/	598.2 ± 47.9	150.2 ± 7.5
	CM	79.1 ± 8.7	83.7 ± 4.2	19.3 ± 1.9	/	156.3 ± 7.8	/	274.4 ± 16.5	/	225.1 ± 18.0	141.9 ± 7.1
	UAE	117.0 ± 12.9	/	13.3 ± 1.3	/	157.1 ± 7.9	/	244.0 ± 14.6	/	516.7 ± 41.3	146.8 ± 7.3
*Pinus heldreichii*	D	86.1 ± 9.5	295.3 ± 14.8	40.0 ± 4.0	/	25.8 ± 1.3	/	197.7 ± 11.9	/	/	74.4 ± 3.7
	CM	49.2 ± 5.4	115.4 ± 5.8	18.5 ± 1.9	/	18.0 ± 0.9	/	186.4 ± 11.2	/	/	88.7 ± 4.4
	UAE	64.1 ± 7.1	177.1 ± 8.9	19.1 ± 1.9	/	16.9 ± 0.8	/	169.0 ± 10.1	/	/	68.5 ± 3.4
*Pinus sylvestris*	D	59.4 ± 6.5	510.5 ± 25.5	12.1 ± 1.2	/	202.9 ± 10.1	/	1563.5 ± 93.8	/	2038.9 ± 163.1	282.1 ± 14.1
	CM	38.3 ± 4.2	935.6 ± 46.8	158.1 ± 15.8	/	148.1 ± 7.4	/	1287.6 ± 77.3	/	1985.2 ± 158.8	239.6 ± 12.0
	UAE	42.7 ± 4.7	901.0 ± 45.0	16.9 ± 1.7	/	187.7 ± 9.4	/	1489.7 ± 89.4	/	2254.7 ± 180.4	511.4 ± 25.6
*Pinus pinea*	D	91.7 ± 10.1	153.9 ± 7.7	6.6 ± 0.7	52.9 ± 3.7	298.0 ± 14.9	/	82.9 ± 5.0	145.9 ± 21.9	69.7 ± 5.6	1450.5 ± 72.5
	CM	90.2 ± 9.9	65.6 ± 3.3	9.5 ± 1.0	55.1 ± 3.9	354.4 ± 17.7	/	99.9 ± 6.0	165.8 ± 24.9	58.5 ±.4.7	1395.9 ± 69.8
	UAE	109.6 ± 12.1	103.7 ± 5.2	103.1 ± 10.3	87.6 ± 6.1	385.7 ± 19.3	/	5.9 ± 0.4	170.2 ± 25.5	78.5 ± 6.3	1537.9 ± 76.9

Values represent mean ± SD of three measurements (*n* = 3). Note: The symbol “/” indicates that the compound was not detected in the analyzed extract.

**Table 7:** (**a**): Antimicrobial activity using (MIC) Minimum Inhibitory Concentration of selected pine species for Gram-positive bacterial strains. (**b**): Antimicrobial activity using (MIC) Minimum Inhibitory Concentration of selected pine species for Gram-negative bacterial strains. (**c**): MIC (mg/mL) of the positive control against the tested bacterial strains (ATCC).

(**a**)				
**Bacteria Strain**		**ATCC**	***Pinus*** **Species**	**MIC—** **Minimum Inhibitory Concentration** **(mg/mL)**
Gram (+)	*Enterococcus faecalis*	29212	*Pinus halepensis*	44
			*Pinus nigra*	44
			*Pinus heldreichii*	44
			*Pinus sylvestris*	66
			*Pinus pinea*	66
	*Enterococcus faecium*	6057	*Pinus halepensis*	29
			*Pinus nigra*	44
			*Pinus heldreichii*	44
			*Pinus sylvestris*	44
			*Pinus pinea*	19
	*Bacillus spizizenii*	6633	*Pinus halepensis*	44
			*Pinus nigra*	44
			*Pinus heldreichii*	66
			*Pinus sylvestris*	19
			*Pinus pinea*	29
	*Staphylococcus aureus*	6538	*Pinus halepensis*	29
			*Pinus nigra*	44
			*Pinus heldreichii*	44
			*Pinus sylvestris*	29
			*Pinus pinea*	44
(**b**)				
**Bacteria Strain**		**ATCC**	***Pinus*** **Species**	**MIC—** **Minimum Inhibitory Concentration** **(mg/mL)**
Gram (−)	*Proteus mirabilis*	25933	*Pinus halepensis*	44
			*Pinus nigra*	44
			*Pinus heldreichii*	66
			*Pinus sylvestris*	44
			*Pinus pinea*	44
	*Escherichia coli*	25922	*Pinus halepensis*	44
			*Pinus nigra*	44
			*Pinus heldreichii*	44
			*Pinus sylvestris*	44
			*Pinus pinea*	66
	*Klebsiella aerogenes*	13048	*Pinus halepensis*	44
			*Pinus nigra*	44
			*Pinus heldreichii*	66
			*Pinus sylvestris*	66
			*Pinus pinea*	44
	*Klebsiella pneumoniae*	13883	*Pinus halepensis*	44
			*Pinus nigra*	44
			*Pinus heldreichii*	44
			*Pinus sylvestris*	44
			*Pinus pinea*	66
	*Pseudomonas aeruginosa*	9027	*Pinus halepensis*	44
			*Pinus nigra*	44
			*Pinus heldreichii*	44
			*Pinus sylvestris*	44
			*Pinus pinea*	29
	*Salmonella enteritidis* (D)	13076	*Pinus halepensis*	29
			*Pinus nigra*	29
			*Pinus heldreichii*	44
			*Pinus sylvestris*	44
			*Pinus pinea*	44
(**c**)				
**Bacterial Strain**		**ATCC**		**MIC (mg/mL)** **Centriaxone (100 mg/mL)**
*Enterococcus faecalis*		29212		50
*Enterococcus faecium*		6057		-
*Bacillus spizizenii*		6633		12.5
*Staphylococcus aureus*		6538		25
*Proteus mirabilis*		25933		12.5
*Escherichia coli*		25922		6.25
*Klebsiella aerogenes*		13048		25
*Klebsiella pneumoniae*		13883		25
*Pseudomonas aeruginosa*		9027		50
*Salmonella enteritidis* (D)		13076		6.25

**Table 8:** An overview of sample labeling.

Pine Species	*Pinus halepensis*	*Pinus nigra*	*Pinus heldreichii*	*Pinus sylvestris*	*Pinus pinea*
Sample	1	2	3	4	5

**Table 9:** (**a**): IC_50_ values (mg/mL) for the samples obtained by digestion (D). (**b**): IC_50_ values (mg/mL) for the samples obtained by ultrasound-assisted extraction (UAE). (**c**): IC_50_ values (mg/mL) for the samples obtained by conventional maceration (CM).

(**a**)					
	1	2	3	4	5
Cells					
HeLa	>32	10.10 ± 0.09	8.91 ± 0.07	8.23 ± 0.05	30.17 ± 0.15
LS 174T	>32	>32	>32	>32	>32
Fem-x	31.15 ± 0.81	>32	>32	>32	30.07 ± 0.06
MRC-5	>32	31.09 ± 0.08	30.75 ± 0.10	29.91 ± 0.12	>32
(**b**)					
	1	2	3	4	5
Cells					
HeLa	12.05 ± 2.07	8.10 ± 0.59	7.43 ± 2.07	8.03 ± 0.65	6.57 ± 0.52
LS 174T	>32	>32	>32	>32	>32
Fem-x	31.15 ± 0.81	>32	>32	31.75 ± 0.03	29.04 ± 0.06
MRC-5	>32	>32	>32	30.23 ± 0.61	>32
(**c**)					
	1	2	3	4	5
Cells					
HeLa	27.22 ± 1.54	6.18 ± 0.29	11.71 ± 0.37	6.29 ± 1.05	12.16 ± 0.95
LS 174T	>32	>32	>32	>32	>32
Fem-x	>32	30.77 ± 0.27	29.07 ± 0.99	31.82 ± 0.16	30.72 ± 0.19
MRC-5	>32	>32	>32	29.91 ± 0.12	>32

IC_50_ values are presented as the mean of three independent experiments ± standard deviation (SD). IC_50_ values are presented as the mean of three independent experiments ± standard deviation (SD). IC_50_ values are presented as the mean of three independent experiments ± standard deviation (SD).

## Data Availability

The original contributions presented in this study are included in the article. Further inquiries can be directed to the corresponding author.
